# Fair allocation of resources in the moral dilemma of triage

**DOI:** 10.3389/fsoc.2025.1570940

**Published:** 2025-05-13

**Authors:** Peer Keßler, Ivar Krumpal

**Affiliations:** ^1^Department Health and Prevention, Institute of Psychology, University of Greifswald, Greifswald, Germany; ^2^Institute of Sociology, Leipzig University, Leipzig, Germany

**Keywords:** triage, allocation, preferences, fairness, conjoint experiment

## Abstract

Against the background of the COVID-19 pandemic that has shaken societies around the world, the debate about fairness of medical allocation decisions is gaining momentum. Studying a sample of a broad international public (*N* = 1,998), we investigate citizens' ethical preferences in the moral dilemma of triage decisions. First, we address the key problem of which of several contradictory ethical criteria and normative principles should be used to determine the fairness of outcomes in triage situations. Preferences about fair outcomes are inferred from observed allocation decisions in a conjoint experiment. Second, preferences in regard to fair procedures are measured via fairness ratings of a series of triage procedures. Third, we analyze the relationship between the observed allocation outcomes and the fairness ratings of procedures. Finally, we review the current expert discourse and reflect it with the citizens ethical preferences observed in our study.

## 1 Introduction

The discussion around the fair allocation of scarce medical resources in triage situations has been a topic of increasing attention during the COVID-19 pandemic (Albertsen, 2023; Brown et al., 2020; Emanuel et al., 2020, 2022; Gradwohl et al., 2024; Knochel et al., 2024; Meier, 2022; Sprengholz et al., 2023; Truog et al., 2020; Tutić et al., 2022; Schmidt and Kriwy, 2022). Triage refers to decisions about allocating medical resources to one patient at the expense of another (Persad et al., 2009). In such an emergency situation careful balancing of highly conflicting normative principles is required. Normative conflicts may arise in triage situations due to the presence of multiple equally important criteria, e.g., the maxim to treat everyone equally may contradict the principle of saving the most lives when the persons in question differ in, for example, age, disability, or weight. Additionally, it is likely that some criteria are only met if others are violated, e.g., the prioritization of patients with a high probability of short-term survival would conflict with other principles, such as benefiting the worst off.

Note that our study focuses on an extreme form of triage, i.e., the decision between life and death in situations of disaster, e.g. wars, terror attacks, or pandemics. Our study does not focus on routine emergency department triage. What is considered fair in the allocation of scarce medical resources depends strongly on the context and the consequences at stake (Hyder, 2020; Krütli et al., 2016; Persad et al., 2009).

In a highly cited commentary, Emanuel et al. (2020) argue that the following ethical principles of resource allocation can be applied in triage situations: (1) The first possible guiding principle is to *maximize benefits*, which means using limited resources in such a way that the overall survival of patients is maximized (“Save the most,” see also Gelinsky, 2020). Consistent with this perspective, it can be justified to stop the treatment of a patient and reallocate medical resources to another patient with a better medical prognosis. *Maximizing prognosis* is often considered the highest priority in triage decisions (Emanuel et al., 2020). For this prognosis, however, different time horizons such as the probability of short-term survival, long-term life-expectancy or even the future quality of life could be considered (Awad et al., 2022). According to Emanuel et al. (2020), the priority of saving as many lives or as many life years as possible can be considered a consensus among ethical experts and across various triage guidelines. In contrast, *maximizing future quality of life* has a lower priority because limited time and information in an emergency situation make an accurate prognosis about future life quality impossible. In fact, the theoretical work of Bognar (2024) argues against using life quality in triage decisions at all, because it is almost impossible to predict future quality of life not only as it relates to health but overall – considering all the aspects of life that determine a patient's long-term life quality. Furthermore, the criterion of life quality is not necessarily related to the probability of short term-survival. Prioritizing life quality in triage decisions would carry the risk of unfairly maximizing the benefits of persons without overweight or without any comorbidities, such as physical disability or dementia (Awad et al., 2022). Such a practice would violate norms of nondiscrimination and could even lead to legal consequences (Parsons and Johal, 2020; Solomon et al., 2020). In general, saving as many lives or as many years of life as possible conflicts with the second conceivable ethical principle of fair resource allocation, the equal treatment of all patients.

(2) *Treating people equally* means that no one should be unfairly discriminated against based on medically irrelevant social categories such as gender or ethnicity. If there are correlations - even unintentional ones - between medically relevant survival probabilities and social categories, the ethical principle of equality would be violated. For example, certain disabilities or overweight may be associated with a generally poorer health status and lower survival probabilities. According to the principle of *treating people equally*, an allocation procedure is considered fair if it leads to outcomes not showing differences in regard to social categories, or if it ensures that everybody has an equal chance to obtain the same outcome. Strict equal treatment would therefore be guaranteed in practice if it were based on selection by random chance (“Random allocation, such as a lottery,” see Emanuel et al., 2020). Alternatively, a strict equality norm could be enforced by not allocating the scarce resources at all (Elster, 1992). Therefore, no one would be treated in a triage situation with two otherwise equal patients. This, in turn, would violate the principle of maximizing benefits by wasting medical resources.

(3) Furthermore, scarce medical resources could be allocated in accordance with the ethical principle to *give priority to the worst off* . The allocation of medical resources to the worst off (“Sickest-first,” see Emanuel et al., 2020) stands in line with the Hippocratic Oath (Markel, 2004). Empathy, the intrinsic value of life, and fundamental rights of human dignity are at the heart of this guiding principle. Withdrawing treatment from someone who is in need without their consent in order to allocate these intensive care resources to a new patient with better prognosis conflicts with two moral notions, i.e., the idea of equality of life and the “sickest first” principle (Emanuel et al., 2020). From a human rights perspective, withdrawing treatment from the sickest patients to save the lives of those with a better prognosis is inherently wrong, irrespective of outcomes (Brown et al., 2020). Additionally, this practice is unlawful in different European countries (Brown et al., 2020). Currently, there is no unified international consensus in regard to the ethical dilemma of withdrawing intensive care in favor of another patient (Gelinsky, 2020).

(4) Another possible principle to allocate scarce medical resources in triage situations is *promoting and rewarding instrumental value* to society (“Benefit to others,” see Emanuel et al., 2020). From a retrospective standpoint, such a value could comprise of prosocial behavior in the past, such as doing volunteer work or getting vaccinated during the COVID-19 pandemic (Zamir and Gillis, 2023). During the COVID-19 pandemic, new norms of prosocial behavior have emerged, such as the norm to get vaccinated to protect others and contribute to the collective good of public health (Berger and Krumpal, 2021). In line with Korn et al. (2020) who argue that vaccination was a social contract and that getting vaccinated is a prosocial behavior benefiting society, it could be argued that the free riding problem could be solved by benefiting norm compliers and sanctioning free riders in triage decisions. From a prospective standpoint, instrumental value to society could consist of future family responsibilities (Daugherty Biddison et al., 2019). It could be argued that caretakers of children are likely to make relevant contributions to society and, therefore, should be prioritized in triage decisions.

The discourse surrounding the fair allocation of scarce medical resources in triage situations remains controversial due to conflicting ethical values and principles involved (Meier, 2022; Sprengholz et al., 2023). Against this background, experts seek to define triage recommendations that aim to achieve a fair allocation of scarce medical resources (Gelinsky, 2020). While there is a worldwide consensus that allocation decisions must be *fair*, there is ethical heterogeneity regarding what outcomes or procedures are considered *fair*. From the perspective of distributive justice (Homans, 1961; Elster, 1992), on the one hand, *outcome fairness* judges the results that are produced by allocation decisions. On the other hand, *procedural fairness* assesses the process and rules used to make the triage decision (Daly and Tripp, 1996). While former empirical research has been focused on either outcome fairness (Tutić et al., 2022; Stoetzer et al., 2023) or procedural fairness (Awad et al., 2022) of triage decisions, little is known about how these two correspond to each other.

On a theoretical level, the relationship between outcome fairness and procedural fairness is far from trivial (Daly and Tripp, 1996). For instance, a citizen might judge an outcome as fair because it is in accordance with her preferences in the situation, e.g., favoring a parent over a person without kids in a triage situation. In turn, this does not imply that the said citizen is in favor of the underlying allocation procedure, e.g., the life-saving resources were allocated to the parent by random lottery. However, if citizens are asked what a fair allocation in a specific triage situation was, the answer can be expected to be backed by an underlying decision rule. Therefore, we expect a close match between fair outcomes and procedures.

Furthermore, public fairness preferences may disagree with ethical fairness criteria defined by experts. Even if a single fairness criterion could be found in advance by experts and used as a normative basis for decisions in triage situations, the fairness preferences of various stakeholders may differ, e.g., the lobby of handicapped patients. In democratic societies, therefore, expert ethics needs to be open for public debate on how to solve the allocation problem in a moral dilemma situation, thereby improving the inclusiveness of the debate (Awad et al., 2018, 2022; Näher et al., 2024).

We seek to shed light on these issues in four ways. First, we measure outcome fairness via stated preferences data from a conjoint experiment. Second, we measure the fairness of procedures via fairness ratings of a series of triage procedures. Third, we shed light on the link between fair outcomes and procedures by relating empirically observed allocation outcomes from the conjoint experiment to the fairness ratings of triage procedures. Finally, we contrast fairness preferences of a broad international public with expert ethics. This allows us to explore the existence of universal norms connecting the expert system and society.

## 2 Materials and methods

### 2.1 Study sample

The participants for this study were recruited via the UK online panel provider Prolific where people can voluntarily register to participate in studies and opinion polls (www.prolific.com). Our survey was fielded in North America and Europe. The study was displayed on the Prolific feeds of all people eligible for our study until the targeted sample size was full. Overall, 2, 282 subjects participated and gave consent for the use of their answers for scientific purposes. To ensure high data quality, we included three attention checks in our survey. We excluded all participants who failed one of the three attention checks or finished the survey in under five minutes. Completing the survey in less than five minutes is impossible for participants who took the survey seriously. This leaves us with the final sample size of *N* = 1, 998 participants. The persons in our sample are on average middle aged (*M* = 35.6, *SD* = 12.8) and completed, on average, 16.3 years of education (*SD* = 3.4). Our sample is slightly skewed toward male-identifying participants. Of all participants in the final sample, 42.1% identify as female and 55.5% as male. 2.4% of the participants chose to describe themselves as diverse and only one person did not enter any gender. For further details on the sampling process, the sampling distribution, and the attention checks, see the Appendix.

### 2.2 Data collection and questionnaire

Data collection of the main study was conducted in August 2023 using SoSci Survey (https://www.soscisurvey.de). All interviews were conducted in English language. Before the main study, the questionnaire was pretested with native English speakers and non-natives, as well as academics and non-academics. After developing a first version of the questions, we conducted qualitative interviews with a small number of laypeople from our social networks (*N* = 5). For the assessment of the questionnaire, we focused on whether respondents understood the questions and instructions. Based on their feedback, the questionnaire was revised and formulations were clarified. Next, we conducted a small pilot study with two groups of undergraduate students from the Leipzig University recruited in a lecture and a research seminar. We identified and corrected errors in conjunction with the wording and sequence of questions, the routing instructions, and the coding structure of the online questionnaire. As part of this pilot-study, the student respondents could comment on the online questionnaire. Based on their feedback, the survey instrument was further improved and finalized.

### 2.3 Survey and conjoint experiment

To evaluate ethical preferences regarding outcome fairness, we integrated a conjoint experiment in the first part of our survey (Hainmueller et al., 2014, 2015). The participants were presented with a fictional scenario stating that they were part of an ethics committee and had to give recommendations in specific triage situations. In these situations, there was only one respiratory ventilator available but needed by two patients. Without ventilation, the patients virtually had no chance of survival. The participants were then asked to decide which one of the two patients should be ventilated with the only ventilator available.

The patients were represented by randomly generated profiles that contained of eight attributes with at least two possible attribute levels for each attribute. These attributes were selected based on the research literature sketched above and were assigned to different ethical principles. The attribute levels were randomized within participants by randomly drawing from a multivariate uniform distribution. Consequently, all variables that refer to patients' attributes are uniformly distributed and statistically independent qua experimental design. However, the experiment was programmed to avoid that the two profiles in each pair are fully equal. Therefore, pairs of profiles differed at least in one of the eight attributes. Additionally, to rule out possible primacy or order effects, the presentation order of attributes was randomized between participants. This fully randomized experimental design allows us to estimate causal average marginal component effects (AMCE) for each attribute which can be interpreted as the probability difference between an attribute level and a reference level to be chosen in the conjoint experiment (Hainmueller et al., 2014).

Each participant was presented with ten pairs of profiles in total. For the first five pairs of profiles the conjoint experiment was designed as a paired conjoint design with forced choice (Hainmueller et al., 2015). That is, for each pair of profiles a participant had to decide which of the two patients should be ventilated. For the last five pairs of profiles, the participants had the additional option to choose a selection of a profile by random chance. That is, for each pair of profiles subjects could actively decide which of the two patients should be ventilated or could opt for a random lottery giving each patient an equal chance of being selected for ventilation. For a more detailed description of the experiment and exemplary pairs of profiles, see the Appendix.

After completing the conjoint experiment, data on preferences about procedural fairness was collected. The fairness of procedures was measured by ratings of eight different allocation procedures on a 6-point scale from 1= “not fair at all” to 6= “very fair.” The procedures were presented with a short title for the procedure and a short description of the procedure. Each procedure specified a rule how the priority should be determined. To avoid spillover and order effects, the order of the procedures on the survey page was randomized. For an overview of the allocation procedures and their corresponding patients' attributes, and underlying ethical principles, see Table 1. For a more detailed description, see the Appendix.

**Table 1:** Overview of allocation procedure with corresponding attribute in the experiment and underlying ethical principle.

**Allocation procedure**	**Attribute in experiment**	**Ethical principle**
*Maximize prognosis*: Prioritize patients with the highest probability of survival after the treatment; i.e., treat those with the highest chance of recovery.	Chance of survival (20% vs. 50% vs. 80%)	*Maximize benefits*(save the most lives)
*Youngest first*: Prioritize younger patients; i.e., treat those who have the most years of life left after overcoming the disease.	*Age* (25 years vs. 50 years vs. 75 years)	*Maximize benefits*(save the most life years)
*Life quality after recovery*: Prioritize patients without any medical preconditions that would reduce quality of life after overcoming the disease; i.e., treat those with the highest quality of life they're likely to have after the treatment.	*Overweight; Disability* (Is not overweight vs. Is overweight; Has no disability vs. Has a disability)	*Maximize benefits*;*Treat people equally*(no discrimination)
*Random selection*: Ventilators should be allocated by random lottery; i.e., individual characteristics should not be considered.	*Random-chance* option	*Treat people equally*(no discrimination)
*First-come, first-served*: Prioritize patients who were first in line; i.e., treat those who arrived first at the hospital.	*Ventilation status* (Not yet ventilated vs. Already ventilated)	*Treat people equally*(no discrimination)
*Sickest first*: Prioritize patients who suffer the most; i.e., treat those who are the worst off.	*Chance of survival* (20% vs. 50% vs. 80%)	*Give priority to the worst off*
*Benefit to others in the past*: Prioritize patients who have made relevant contributions to the benefit of others; i.e., treat those who have made sacrifices helping with the virus by having themselves vaccinated.	*Vaccination status; Volunteer work* (Is not vaccinated vs. Is vaccinated; Does not volunteer work vs. Does volunteer work)	*Reward instrumental value to others*
*Benefit to others in the future*: Prioritize patients who are likely to make relevant contributions to the benefit of others; i.e., treat those who raise children.	*Children* (Has no Children vs. Has Children)	*Reward instrumental value to others*

For the analysis of the conjoint experiment, we followed the approach by Hainmueller et al. (2014). The causal AMCEs were calculated by using a linear probability model with clustered standard errors on the participant level to account for the multilevel structure of the data. The dependent variable in this model is represented by the choice of a profile while the attributes of the profiles are used as independent variables.

Finally, heterogeneous causal effects of patients' attributes conditional on the corresponding fairness ratings of procedures were estimated via including interaction terms between attributes and ratings in our statistical model. In a preceding step, we dichotomized the ratings in *not fair* (scores lower than four) and *fair* (scores greater or equal four). Significant interaction effects indicate an association between fairness ratings of procedures and outcomes in our conjoint experiment.

To evaluate the link between the choice of a selection by random chance and the fairness rating of the “random selection”—procedure, the correlation (Kendalls' Tau) of the relative frequency of choosing *random chance* and the fairness rating was calculated. Since there are only five pairs of profiles with the random-chance option, the relative frequency could only take the numbers 0.2, 0.4, 0.6, 0.8, and 1. Additionally, we used graphical analysis to evaluate the correlation. We used a jitter plot and calculated a bivariate linear OLS regression for the prediction line of the relative frequency of choosing a selection by random chance given the procedure rating of a random allocation.

## 3 Results

### 3.1 Effects of attributes in the conjoint experiment

**Figure 1:**
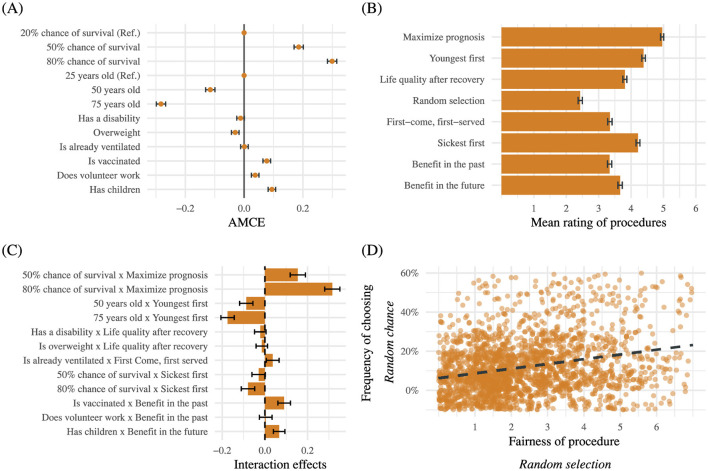
The graphs show the results of the conjoint experiment and the procedure ratings. Bars in all graphs represent 95% confidence intervals. **(A)** The graph shows the AMCEs for all attributes in the conjoint experiment based on all pairs of profiles without the *random-chance* option. The graph is based on *N* = (5 × 2 × 1, 998) = 19, 980 data points that are clustered in 1, 998 participants. The confidence intervals are based on clustered standard errors on participant level to account for the multi-level structure of the data. **(B)** The graph depicts the mean rating of procedures (1 = Not fair at all, 6 = Very fair). The graph is based on *N* = 1, 998 data points. **(C)** The graph shows the interaction terms between the attributes in the conjoint experiment and the dichotomized ratings of corresponding allocation procedures. The reference category for the dichotomized ratings is *not fair* (scores lower than 4). The graph is based on *N* = (5 × 2 × 1, 998) = 19, 980 data points that are clustered in 1, 998 participants. **(D)** The Graph shows the bivariate linear regression between fairness ratings of the *Random selection*-procedure and the relative frequencies of choosing the allocation by random chance. The predicted values of the relative frequencies are represented by the dotted line. The relative frequency is based on all pairs of profiles with the *random-chance* option. The graph is based on *N* = 1, 998 data points.

Figure 1A visualizes the results of the allocation decisions regarding the five pairs of profiles without the random-chance option in our conjoint experiment (for the corresponding regression table, see the Appendix). It shows the estimates of the AMCEs for all patients' attributes on the probability of getting ventilation. The depicted 95% confidence intervals were calculated based on clustered standard errors, which account for the clustering of choices within participants. The strongest effects show for the attributes *Chance of survival* and *Age*: on average, a patient with an 80% chance of survival has a 29.9% higher probability of receiving ventilation compared to a patient with a 20% chance of survival. Similarly, a patient with a 50% chance of survival has an 18.6% higher probability of receiving ventilation compared to a patient with a 20% chance of survival. Furthermore, participants choose younger patients significantly more often than older patients. Seventy five-year-old patients have a 28.3% and 50-year-old patients have a 11.5% lower probability of getting ventilation in comparison to 25-years-old patients. For the patients' ventilation status, in contrast, we find a null effect. These results can be interpreted as allocation decisions consistent with the approach to *Maximize benefits*. At the same time, participants clearly reject the *First-come-first-served* principle.

In contrast to the strong effects of the attributes *Chance of survival* or *Age*, significantly positive but less pronounced effects can be observed for attributes reflecting social issues: patients who are vaccinated (7.7%), have children (9.4%), or do volunteer work (3.8%) have on average a somewhat higher probability of getting ventilation than persons from the respective references groups. These findings speak in favor of some *rewarding of prosocial behavior* and of *instrumental value* in a society.

Finally, although significantly different from zero, the effect size of the attribute *Overweight* (−3%) is substantially small and close to zero and the effect of the attribute *Disability* (−1%) is even smaller and statistically insignificant. We interpret these results as general support for the existence of norms of non-discrimination prescribing that nobody should be discriminated unfairly based on criteria other than medical prognosis.

### 3.2 Fairness-ratings of triage procedures

Figure 1B shows the results for the fairness-ratings of allocation procedures. Note that the descriptive analyses in this subsection refer to the level of respondents, not to the level of decisions. “Maximize prognosis,” i.e., prioritizing patients with the greatest chance of survival, was on average rated as the fairest procedure (*M* = 5.0, *SD* = 1.1). The “Youngest first”—procedure, i.e., prioritizing younger over older patients, was rated as second fairest (*M* = 4.4, *SD* = 1.3), closely followed by the “Sickest first”—procedure, i.e., the prioritization of patients who suffer the most (*M* = 4.2, *SD* = 1.3). On average, the respondents rated the “Random selection”—procedure, i.e., ventilators should be allocated by random lottery, as at least fair (*M* = 2.4, *SD* = 1.5). The fairness ratings of the remaining procedures were similar and located between these extreme values: “First-come, first served” (*M* = 3.3, *SD* = 1.5), “Benefits to others in the past” (*M* = 3.3, *SD* = 1.5), “Benefits to others in the future” (*M* = 3.7, *SD* = 1.5), and the prioritization of “Life quality after recovery” (*M* = 3.8, *SD* = 1.4). These findings underscore the picture that citizens fairness preferences align with the ethical principle to *maximize benefits* in triage situations. At the same time, citizens clearly reject allocating resources by means of a random lottery, i.e., implementing a strict equality norm and ignoring individual characteristics completely is judged unfair.

### 3.3 Interaction effects between attributes and fairness ratings

To study the connection between fair outcomes and procedures, interaction effects between the attributes from the conjoint experiment and the fairness ratings of the corresponding allocation procedures were estimated. Figure 1C visualizes the results regarding the five pairs of profiles in the conjoint experiment without the random-chance option (for the regression tables of the model, see the Appendix). For simplicity, fairness ratings were dichotomized (values over and equal 4 into *fair* and values lower than 4 into *not fair*). The visualized interaction effects can be interpreted as differences in effect sizes of the attributes in the conjoint experiment between subjects who rate the corresponding procedure as *fair* and subjects who rate the procedure as *not fair* (the reference group).

We find the largest differences in effect sizes for the attributes *Chance of survival* and *Age*: In comparison to the reference group, respondents who rate the “Maximize prognosis” procedure as fair show a significantly stronger reaction to variations in the corresponding attribute *Chance of survival* (50% chance of survival × Maximize prognosis: *D* = 15.4%, *p* < 0.001; 80% chance of survival × Maximize prognosis: *D* = 31.6%, *p* < 0.001). In other words, subjects who rate the procedure as *fair* placed much greater weight in their allocation decision on the corresponding attribute *Chance of survival* than respondents who rated the procedure as *unfair*.

Since the attribute *Chance of survival* also corresponds with the “Sickest first” procedure, these interaction terms were also included in our statistical model. Participants who evaluate the “Sickest first”—procedure as *fair* give significantly lower priority to patients with an 80% chance of survival compared to subjects in the reference group (80% chance of survival × Sickest first: *D* = −7.9%, *p* < 0.001). For patients with a 50% chance of survival, we do not find a significant interaction effect (50% chance of survival × Sickest first: *D* = −2.9%, *p* = 0.07). Still, the sign of the effect points in the expected direction.

Furthermore, in comparison to subjects who rated the “Youngest first”—procedure as *unfair*, subjects who rated the “Youngest first”—procedure as *fair* showed a significant increase in the effect size of the attribute *Age* (50 years old × Youngest first: *D* = −8.7%, *p* < 0.001; 75 years old × Youngest first: *D* = −17.5%, *p* < 0.001). The direction of the effect size changes is as theoretically expected, i.e., subjects who rate the procedure as *fair* give younger patients higher priority in their allocation decisions compared to the reference group.

All other significant interaction effects are rather small: Is already ventilated × First come, first served (*D* = 3.7%, *p* = 0.02), Is vaccinated × Benefit in the past (*D* = 9.0%, *p* < 0.001), and Children × Benefit in the future (*D* = 6.7%, *p* < 0.001). This finding is not surprising given the rather small effects of the specific attributes in the experiment. Still, the effects show the projected sign.

Note that the remaining interaction effects are not significant: Has a disability × Life quality after recovery (*D* = −2.2%, *p* = 0.12), Is overweight × Life quality after recovery (*D* = −1.4%, *p* = 0.29), and Does volunteer work x Benefit in the past (*D* = 0.4%, *p* = 0.79). These results can be interpreted as evidence for a consensus among the participants to evaluate these medically irrelevant attributes independently of the rating of the corresponding procedures. More specifically, the results speak in favor of strong anti-discrimination norms among the participants.

We tested the robustness of these results using different integrations of the procedure ratings. Namely, we repeated the same analyses with the ratings dichotomized by using the median of the rating as the split value. In addition, we included the ratings as numerical values in the model. Both integrations produced similar results (see the Appendix).

### 3.4 The fairness of a random lottery

Recall that for the last five pairs of profiles in our conjoint experiment, participants had the additional option to choose a selection of a profile by *random chance*. That is, for each pair of profiles, subjects could actively decide which of the two patients should be ventilated, or could opt for a random lottery giving each patient an equal chance of being selected for ventilation. Overall, participants chose an active allocation decision instead of delegating the decision to *random chance* in the majority of the pairs of profiles. More specifically, the selection by *random chance* was chosen in 20.3% of all pairs of profiles. However, a narrow majority of participants chose the option at least once (61.6%).

In this subsection, we analyze the relationship between the fairness ratings of the “Random selection”—procedure and the relative frequency of choosing the *random-chance* option in the second part of our conjoint experiment. The analyses were calculated on the respondents' level. Figure 1D depicts a jitter plot between fairness ratings and the relative frequencies of choosing the allocation by random chance. The dotted line represents the predicted values of the relative frequencies based on a bivariate linear OLS regression. The visualized data clearly indicate a significant positive relationship between the fairness rating and the choice of the random-chance option (τ_*Kendall*_ = 0.2; *p* < 0.001). That is, respondents who rated the “random selection”—procedure as *fair* chose a selection by *random chance* more often than respondents who rated the procedure as unfair. Our findings are further evidence for the link between fair outcomes and procedures.

## 4 Discussion

Our study contributes to explicating and empirically investigating the normative foundations of what constitutes the fairness of allocation decisions in triage situations, initiates public dialogue on ethical fairness criteria and clarifies the link between fair outcomes and fair procedures (Emanuel et al., 2020). Our results correspond to the hypothesized connection between fair outcomes (Tutić et al., 2022; Stoetzer et al., 2023) and fair procedures (Awad et al., 2022).

First, preferences of outcome fairness were measured in a conjoint experiment on allocation decisions in triage situations. By and large, we find that the observed allocation decisions strongly support the guiding principle to *Maximize benefits*. That is, among all attributes under consideration, the short-term survival chance (reflecting the maxim to “Save the most lives”) and the long-term life expectancy (measured by age; reflecting the principle to “Save the most life-years”) indicate the strongest effects on allocation outcomes in our conjoint experiment. This result is backed by the study of Wilkinson et al. (2020) who, despite using a somewhat different research design, also found strong evidence for the ethical principle of maximizing benefits, prioritizing patients with a higher chance of survival and lower age. In contrast, whether the patient is already ventilated or not carries zero weight in the triage decisions observed in our data. This result supports citizens preferences with respect to the ethical principle of maximizing benefits. However, some experts argue that decision-making consistent with the *Maximize-benefits* approach conflicts with the fundamental rights of human dignity and the ethical principle to *give priority to the worst off* (Brown et al., 2020). With respect to the impact of the attributes *Overweight* and *Disability* on triage decisions, the estimated effect sizes are very small and close to zero. This result supports the principle of *Treating people equally* and indicates that citizens prefer that nobody should be discriminated unfairly based on medically irrelevant criteria. Finally, citizens attach some importance to the principle of *Rewarding and promoting instrumental value* to society. In particular, we observe that attributes reflecting social issues, i.e., being vaccinated, having children or having done volunteer work in the past, have some impact on triage decisions in our study. Thus, prosocial behavior matters in citizens triage decisions. Relating triage decisions to the vaccination status and other forms of prosocial behavior has been part of the public debate in the context of the COVID-19 pandemic (Shaw, 2022). However, most experts would agree that prosocial behavior should not be used as a triage criterion in a medical emergency (Gelinsky, 2020). Note that the effects of attributes reflecting social issues are substantially smaller compared to the effects of *Chance of survival* or *Age* which are consistent with the ethical principle to *Maximize benefits* (Emanuel et al., 2020). Overall, we find that citizens conform relatively well with international triage recommendations in prioritizing the short-term survival chance and the long-term life expectancy in their allocation decisions (Gelinsky, 2020). In addition, our results support the theory of triage by Bognar (2024), who argues that the normative goal of triage should be to maximize life years, constrained by principles of non-discrimination. The findings of this study are consistent with guiding principles of triage in other contexts. For instance, the START procedure has been developed to assess patients' need for treatment when it is impracticable to transport all patients into a medical facility immediately (Benson et al., 1996; Cone and MacMillan, 2005). The objective of this procedure is to ensure the optimal use of available resources consistent with the principle of maximizing benefits. This is primarily achieved by prioritizing the probability of survival, age, and the overall health status as assessment criteria (Benson et al., 1996). Future studies are invited to replicate our study for on-site triage scenario to assess fairness preferences in this context.

Second, preferences in regard to fair procedures were measured via fairness ratings of a series of triage procedures. By and large, we find that the observed fairness ratings strongly support the ethical principle to *Maximize benefits*. Out of all evaluated allocation procedures, prioritizing patients with the highest probability of survival (“Maximize prognosis”) and younger patients (“Youngest first”) received the highest average fairness scores. Interestingly, the observed fairness ratings also support the “Sickest first”—procedure. Prioritizing patients who suffer the most (“Sickest first”) is in accordance with the ethical principle to *give priority to the worst off* . In contrast, procedures reflecting the principle of *Rewarding and promoting instrumental value* to society received lower average fairness ratings (“Benefit in the future;” “Benefit in the past”). Finally, we find clear evidence against the norm of *Treating people equally*. Recall that strict equal treatment would be guaranteed if the triage procedure was based on a random lottery. However, allocating ventilators by random lottery and ignoring individual characteristics completely was clearly rated as at least fair. Rating “Random selection” and “First-come, first-served” as the unfairest procedures matches with international triage recommendations (Gelinsky, 2020). Note that Emanuel et al. (2020) argue for the consideration of a random allocation for patients with a similar prognosis to achieve equality.

In summary, citizens' preferences match expert ethics in prioritizing procedures consistent with the ethical principle to *Maximize benefits*. At the same time, the overall picture of preferences regarding fair procedures is nuanced and the difficult task of integrating contradictory ethical principles and paradoxes is reflected in the citizens' fairness ratings elicited in our study.

Third, we analyze the relationship between allocation outcomes and fairness ratings of allocation procedures. Empirically, fairness preferences for specific allocation procedures substantially increase the effects of attributes representing the same ethical principle in our experiment. For all interaction effects displayed in Figure 1C, the direction of changes in effect sizes is as can be theoretically expected. In summary, our results confirm the link between fair outcomes and procedures showing that citizens decisions are backed by underlying decision rules.

With our sample we focus on the allocation of medical resources in western countries, i.e., North America and Europe. However, Awad et al. (2018, 2020, 2022) found evidence for considerable cross-cultural variation regarding the preferred solutions to various moral dilemmas. For example, differences in ethical values, particularly between collectivist and individualist cultures, are expected to result in different cultural clusters with different decision-making patterns (Rhim, 2020). In order to investigate universals and variations in human morality on a global scale, future research studies are invited to replicate our research design in other geographical regions. Furthermore, moral decision-making may be influenced by respondents' individual characteristics such as age or the stress level experienced during the pandemic (Mazza et al., 2020; Borhany et al., 2023). Future studies could investigate interaction effects between patients' attributes and respondents' characteristics on moral decision making. For example, it could be hypothesized that older respondents are likely to prioritize older patients in fictional triage situations (such as conjoint experiments) because of own-group preferences.

In our study, we focus on allocation decisions of citizens. We find evidence for a good match between expert ethics and public morality. However, some notable differences between expert ethics and public morality can be observed. In their allocation decisions, citizens did not consider a patients' ventilation status, even though this attribute receives much more attention in the research literature on the problem of withdrawing treatment from a patient to reallocate a limited resource to another patient with a better medical prognosis. A possible extension of our research is to replicate our conjoint experiment and collect data on the decision-making patterns of different status groups in the medical system. Thus, more could be learned about the factors explaining the differences between experts' and citizens' ethical preferences regarding the solution of the allocation dilemma. We hope that our work contributes to a global dialogue to achieve a consensus on possible global standards for resolving moral dilemmas such as triage situation. The solution of such dilemmas can only be overcome through the joint efforts of different academic disciplines and the inclusion of different stakeholder groups and the general public in that dialogue.

Furthermore, we think that conjoint analysis, with its merits from a causal-analytical point of view is a stimulating research approach to study ethical preferences and how humans simultaneously balance and weigh multiple conflicting (or sometimes even incompatible) allocation criteria in their decisions in moral dilemma situations such as triage. Finally, we would like to discuss the strengths and limitations of conjoint experiments to study triage decisions. Conjoint experiments have been used in diverse academic and non-academic fields to study normative orientations and decision behavior, including sociology, political science, and psychology (for an overview see Auspurg and Hinz, 2015; Hainmueller et al., 2015, 2014). In addition, conjoint analysis has been applied to study ethical preferences and decision behavior in moral dilemmas, including transportation research (Awad et al., 2018) and health decision-making (Tutić et al., 2022; Stoetzer et al., 2023). The strength of conjoint experiments is a high internal validity, i.e., it is possible to identify and estimate causal effects (AMCEs) of a single attribute, e.g., the chance of survival, independent of the other attributes and compare effect strengths of attributes in triage decisions. However, an inherent limitation of conjoint experiments is that they investigate choice behavior in fictious moral dilemma situations and that the choice behavior observed in the context of conjoint experiments might differ from decisions in the real-world (Tutić et al., 2022). Our triage scenario is not realistic in the sense that it does not capture all possible nuances and aspects of a “real” triage situation or complex triage policies. Rather, it presents a simplified and abstract model of reality to the respondents. In real-life triage situations, certain criteria, such as the chance of survival or life quality, are uncertain and difficult to assess (Awad et al., 2022; Bognar, 2024). In our hypothetical triage scenarios, uncertainty is reduced to a handful of attributes and decision parameters. Furthermore, attributes that are statistically independent in our experimental design are often correlated in real-life situations; e.g. overweight or certain disabilities may be associated with a generally poorer health status and lower survival chances. Moreover, triage decisions are extreme choice situations, in which high stakes are at line, and not representative for the everyday conduct of our nonexpert respondents. How actual choices in real-life emergency situations are made and which other aspects determine allocation decisions could be investigated via field observations and qualitative studies. Such field studies could help to assess the degree of transportability of our experimental findings to real world situations (Bader et al., 2021) and compare decision behavior in hypothetical conjoint experiments with behavioral benchmarks in the real world (Hainmueller et al., 2015). The complementarity of different research approaches contributes more to external validity and generalizability than does any single research method or setting alone (Auspurg and Hinz, 2015). Future research is invited to replicate and compare our results across different contexts, methods and study samples to learn about decision behavior in moral dilemmas.

## Data Availability

The datasets presented in this study can be found in online repositories. The names of the repository/repositories and accession number(s) can be found below: https://github.com/Peer-Kessler/fair_allocation_triage.
