# Supplementary material for: Fair allocation of resources in the moral dilemma of triage
Source: Front Sociol. 2025 May 13;10:1570940. doi: 10.3389/fsoc.2025.1570940 (PMC12106452; doi:10.3389/fsoc.2025.1570940)
Supplement: Supplementary file 1 [file Presentation_1.pdf]

Appendix for

Fair allocation of resources in the moral dilemma of triage

Peer Keßler and Ivar Krumpal

Ivar Krumpal
E-mail: krumpal@sozio.uni-leipzig.de

The PDF file includes
Supporting Text
Figures S1 to S4
Tables S1 to S3
SI References

The supporting information is organized as follows. After the data availability statement we present our survey design in greater detail. The last section contains the survey questions used in our study in the order in which they appeared in our survey. Additionally, we provide the regression table for the results depicted in Fig. 1a), Fig. 1c) and for the robustness checks (see table S3).

Data availability statement

The data analyses were conducted in R. The data set, our main analysis file, and the codebook can be accessed via our GitHub repository (https://github.com/Peer-Kessler/fair_allocation_triage).

Methods

Sample

Study participants from North America and Europe were recruited via the online panel provider Prolific (www.prolific.com). Since subjects from the United Kingdom and the United States are over-represented in the Prolific online panel, we set a limit for participants from these countries (100 for the UK, 800 for the US). For our study, the target sample size was 2000 participants (1000 from North America, 1000 from Europe). Our study was displayed on the Prolific feeds of all people eligible for our study until the targeted sample size was full. Overall, 2282 subjects participated and gave consent for the use of their answers for scientific purposes. Note that our gross sample exceeds the targeted size of 2000 participants. We included anti-bot and attention check questions in our survey (see next section). We excluded all participants who failed any of the attention check questions from the study. The failed participants' slots were vacant in the sample and reopened for other participants. We also excluded anyone who completed the survey in less than five minutes prior to the analysis. Completing the survey in less than five minutes is impossible for participants who took the survey seriously. This is especially the case since the situation description in the experimental part was shown for 30 seconds before participants could continue with the survey. This leaves us with the final sample size of $N = 1998$ participants.

The subjects in our sample are on average middle aged ($M = 35.6$, $SD = 12.8$) and completed, on average, 16.3 years of education ($SD = 3.4$). Our sample is slightly skewed towards male-identifying participants. Of all participants in the final sample, 42.1% identify as female and 55.5% as male. 2.4% of the participants chose to describe themselves as diverse. Only one person did not enter any gender. The participants' distribution across country of residence can be found in table S1. Most participants reside in the United States followed by Portugal, Poland and Canada. One participant reported to live in the United Arab Emirates and one participant did not provide a country of residence. Both cases were kept in the final sample.

All interviews were conducted in English. Therefore, the study sample might be biased towards more educated participants. However, the whole survey was pretested with native English speakers and non-natives, as well as academics and non-academics. After adjustments, the survey texts and procedures were found to be understandable by all participants in the pretest.

Attention checks

As noted before, we used anti-bot and attention-check questions to ensure high data quality which were integrated at three positions of our online questionnaire (beginning, middle and end). More specifically, we incorporated three known types of attention checks[1]: a questions to which everyone knows the answer[2], an instructional manipulation check where the correct answer is hidden in the instructional text[3], and a question giving direct instructions to answer in a specific way[4]. For the first type we simply asked participants to answer the following question: *What shape has a red circle?*. Subjects had to choose between the options *Rectangular*, *Round*, *Green*, and *Red*. Everyone who did not tick *Round* was excluded from the survey.

The second type of question consisted of the following instruction text which was presented as an image to reduce the risks of bot answers:

> *Recent research on decision making shows that choices are affected by context. Differences in how people feel, their previous knowledge and experience, and their environment can affect choices. We are interested in whether you actually take the time to read the directions. To show that you have read the instructions, please ignore the question below about how you are feeling and instead check the None of these option as your answer and continue. Thank you very much.*
> *Please check the word that describes best how you are currently feeling.*

Under the instruction text, participants had to choose between the answer options *Afraid*, *Exited*, *Concerned*, *None of these*. Only participants choosing *None of these* were able to continue. Finally, for the third type of attention-check questions participants were simply asked to select *I fully agree* on a 6-point rating scale in order to indicate that they were paying attention to the content of the questions.

Table S1. Sample by self-reported country of residence.

Country	n	%
Austria	2	0.10
Belgium	12	0.60
Canada	202	10.11
Caribbean Netherlands	1	0.05
Czech Republic	19	0.95
Denmark	6	0.30
Finland	11	0.55
France	29	1.45
Germany	12	0.60
Greece	61	3.05
Hungary	34	1.70
Ireland	17	0.85
Italy	112	5.61
Netherlands	22	1.10
Norway	3	0.15
Poland	224	11.21
Portugal	252	12.61
Spain	82	4.10
Sweden	4	0.20
Switzerland	2	0.10
United Arab Emirates	1	0.05
United Kingdom	100	5.00
United States	787	39.39
United States Minor Outlying Islands	2	0.10
Missing	1	0.05
Sum	1998	100.00

Conjoint experiment

As part of the conjoint experiment the participants were given the following introductory text:

> *Please imagine the following situation:*
> *A pandemic has been spreading for several months. The virus is transmitted by droplets in the air. Vaccination is accessible to all; it is voluntary and protects others as well. It also reduces the risk of serious illness for the vaccinated person himself. After a steep rise in infections, the number of emergency patients with life threatening respiratory conditions is increasing. The national health care system has reached its limits. There are more patients than ventilators. Physicians must now decide who should and who should not be ventilated or kept on a ventilator. Patients who are not ventilated have effectively no chance of survival and can only be given pain relief. Imagine that you are a member of a hospital ethics committee and that you are advising the physicians in the intensive care unit on these difficult and stressful decisions. In the following, you will be presented with different situations, one after the other. In each of these situations there are two patients but only one available ventilator. The second ventilator is to be moved to another room in the intensive care unit where it is urgently needed.*
> *After 30 seconds you can proceed with the survey.*

At the bottom of the introduction page a timer, which was counting seconds backwards from 30 seconds to 0 seconds, was shown. After the 30 seconds expired, the participants were able to continue with the study.

The conjoint experiment itself consisted of two sections. In each section participants were presented with five pairs of profiles (ten pairs in total). The first section used a paired conjoint design with forced choice, i.e., for each pair of profiles, participants had to choose between one of the two patients profiles (for an example see Fig. S1). In the second section participants had the additional option to choose a selection of a profile by *Random chance* giving each patient an equal chance of being selected for ventilation (for an example see Fig. S2). The patients' profiles consisted of eight attributes which were randomly drawn from a multivariate uniform distribution. Consequently, all variables that refer to patients' attributes are uniformly distributed and statistically independent qua experimental design[5]. However, the occurrence of two identical profiles in a pair of profiles was suppressed in the programming. Therefore, the profiles always differed in at least one attribute. Additionally, the order in which

the attributes were presented was randomized between participants and was held constant for the whole conjoint experiment. An overview of all attributes and the corresponding attribute levels in our conjoint experiment can be found in Table S2.

Please choose which person should be ventilated.

Please review the information on both patients thoroughly and make a recommendation.

	**Person A**	**Person B**
**Age**	50 years old	75 years old
**Weight**	Overweight	Not overweight
**Volunteer work**	Does volunteer work	Does not volunteer work
**Vaccination status**	Is vaccinated	Is vaccinated
**Children**	Has no children	Has no children
**Chance of survival with ventilation**	20% chance of survival	50% chance of survival
**Disability**	Has no disability	Has no disability
**Ventilation status**	Not ventilated yet	Is already ventilated

○ Person A ○ Person B

Figure S1. Example for a pair of profiles in the forced-choice design.

Please choose which person should be ventilated or leave the selection to random chance.

Please review the information on both patients thoroughly and make a recommendation.

	**Person A**	**Person B**
**Age**	75 years old	25 years old
**Weight**	Not overweight	Overweight
**Volunteer work**	Does volunteer work	Does not volunteer work
**Vaccination status**	Is vaccinated	Is vaccinated
**Children**	Has children	Has children
**Chance of survival with ventilation**	80% chance of survival	20% chance of survival
**Disability**	Has a disability	Has a disability
**Ventilation status**	Is already ventilated	Is already ventilated

○ Person A ○ Person B ○ Random Chance

Figure S2. Example for a pair of profiles with an additional *Random-Chance* option.

Table S2. Attributes and Attribute Levels in conjoint experiment.

Attribute	Attribute Level
Chance of survival with ventilation	20% chance of survival
	50% chance of survival
	80% chance of survival
Age	25 years old
	50 years old
	75 years old
Disability	Has no disability
	Has a disability
Weight	Not overweight
	Overweight
Ventilation status	Is not yet ventilated
	Is already ventilated
Vaccination status	Is not vaccinated
	Is vaccinated
Volunteer work	Does not volunteer work
	Does volunteer work
Children	Has no children
	Has children

Triage Procedures

Subsequent to the conjoint experiment, participants were asked to rate how morally fair they think eight different allocation procedures are on a six-point scale from 1 = "*not fair at all*" to 6= "*very fair*". The rating task was introduced by the following text:

> *In a pandemic, such as COVID-19, many hospitals face situations where the demand for ventilators among patients in need will exceed the number of ventilators that are available. In that case, difficult decisions, just like the ones you just made, will need to be made about who should receive one of the scarce ventilators available. In the following, you see different procedures on how to determine the priority that patients have for being allocated a ventilator.*
> *Please indicate how morally fair you find the following procedures.*

To avoid spill-over and order effects, the order of the procedures was randomized on the survey page.

Robustness checks

We tested our results for robustness regarding the dichotomization of the fairness ratings. First, we repeated our analyses with the median split of the fairness ratings instead dichotomization by using the scale midpoint (between 3 and 4). As can be seen in Fig. S3 the analyses gives the same results as Fig. 1c) in the main article. The same holds true if the fairness ratings are included as numeric values in the model (see Fig. S4). All data analyses were done using unweighted data.

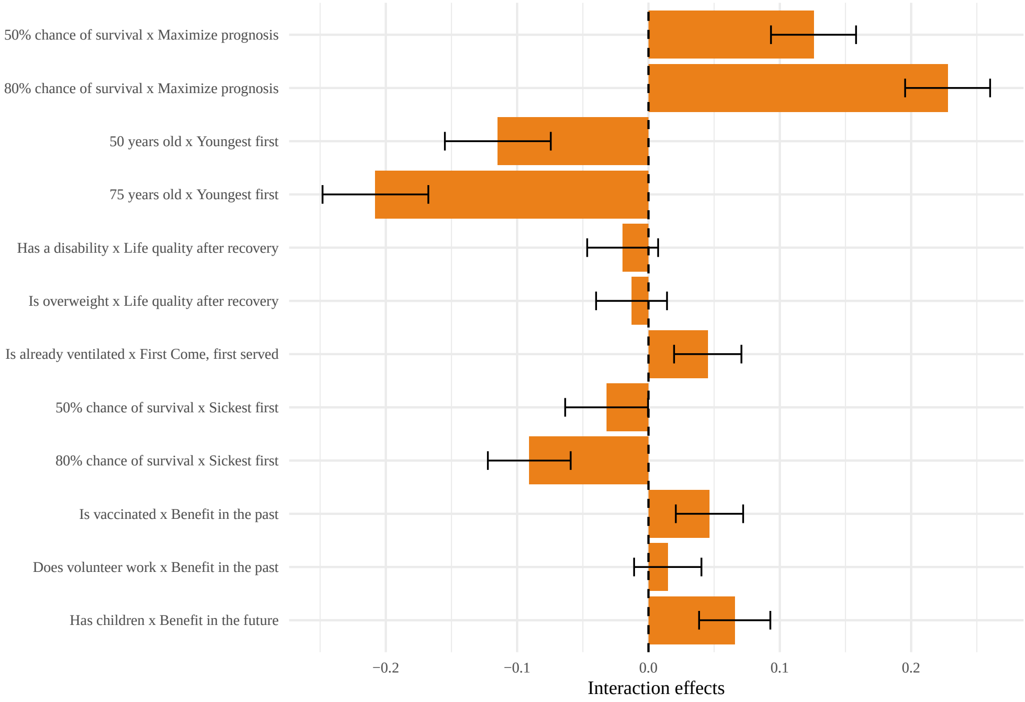

Figure S3. Robustness check with median split for procedure rating.

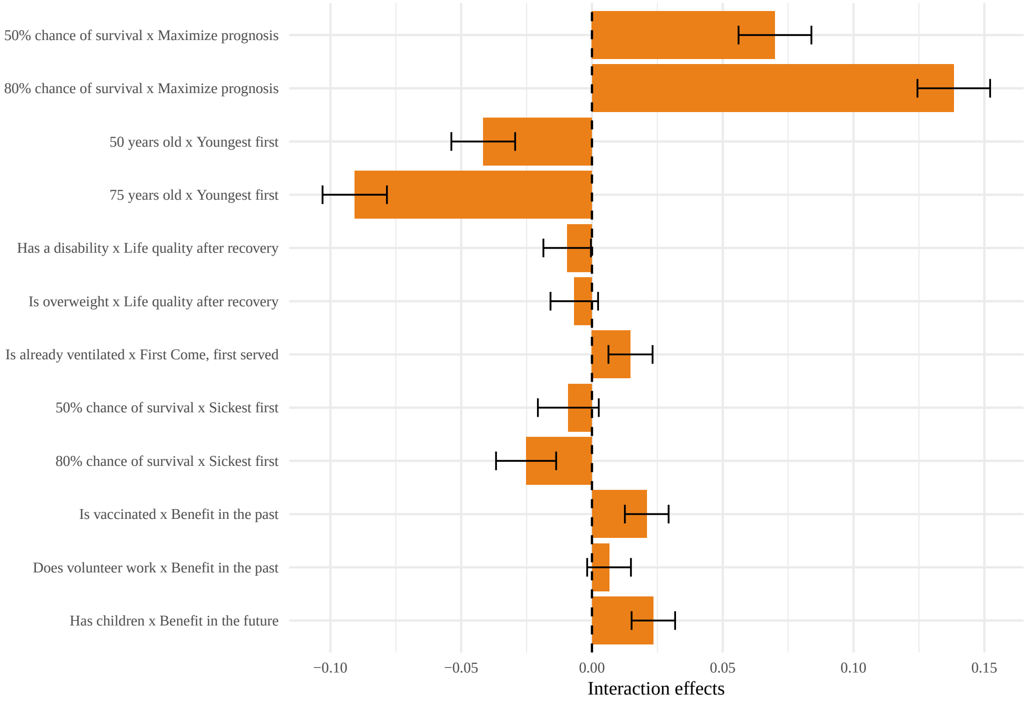

Figure S4. Robustness check with numeric values for procedure rating.

Documentation of the survey questions

The following describes all survey questions we used for our data analyses in the order in which they appeared in the survey.

Introduction:

Dear Participant,

the Institute of Sociology at the University of Leipzig, Germany, is conducting a population survey. We are interested in your opinion about medical care in society.

Researcher: PD Dr. Ivar Krumpal, University of Leipzig; Peer Keßler M.A., University of Leipzig.

This research project is funded by the German Research Foundation.

Answering the questions in this survey will take about 20 minutes.

Your participation is voluntary. All your data is anonymous and subject to data protection. Once the survey is completed, you will be given a payout code that you can use to get paid by Prolific.

Please read all instructions and questions carefully and answer honestly. To ensure data quality we included attention checks in the questionaire. Failing the attention checks will automatically end the survey and you will not be paid.

Please decide now if you want to give your consent to the use of your answers for scientific purposes.
If you do not agree, you will be redirected to the end of the survey and you will not be paid.

☐ I consent to the use of my data submitted as part of this questionnaire for research purposes.
☐ I do not consent to the use of my data submitted as part of this questionnaire for research purposes.

Bot question:

What is the shape of a red circle?
Please provide the correct answer. This is a quality question to identify bots.

☐ Rectangular
☐ Round
☐ Green
☐ Red

Attention check question 1:

Recent research on decision making shows that choices are affected by context. Differences in how people feel, their previous knowledge and experience, and their environment can affect choices. We are interested in whether you actually take the time to read the directions. To show that you have read the instructions, please ignore the question below about how you are feeling and instead check the None of these option as your answer and continue. Thank you very much.
Please check the word that describes best how you are currently feeling.

☐ Afraid
☐ Exited
☐ None of these
☐ Concerned

Introduction to the conjoint experiment:

On the next page, you will see a description of a fictitious situation.

Read the entire text in detail. Do not leave anything out. Then put yourself in this situation. To make sure that you take your time reading the description, you can continue with the survey after 30 seconds.

Afterwards, you need to make some decisions in this situation.

Situation description:

Please imagine the following situation:

A pandemic has been spreading for several months. The virus is transmitted by droplets in the air. Vaccination is accessible to all; it is voluntary and protects others as well. It also reduces the risk of serious illness for the vaccinated person himself. After a steep rise in infections, the number of emergency patients with life-threatening respiratory conditions is increasing.

The national health care system has reached its limits. There are more patients than ventilators.
Physicians must now decide who should and who should not be ventilated or kept on a ventilator. Patients who are not ventilated have effectively no chance of survival and can only be given pain relief.

Imagine that you are a member of a hospital ethics committee and that you are advising the physicians in the intensive care unit on these difficult and stressful decisions.

In the following, you will be presented with different situations, one after the other. In each of these situations there are two patients but only one available ventilator. The second ventilator is to be moved to another room in the intensive care unit where it is urgently needed.

After 30 seconds you can proceed with the survey.

X seconds

Conjoint experiment without random-chance option; every participant was given 5 of these pairs of profiles. Since the pairs were fully randomized, a generalized version is presented here (for a specific example see also fig. S1).

Please choose which person should be ventilated.
Please review the information on both patients thoroughly and make a recommendation.

	Person A	Person B
Attribute 1	Attribute Level	Attribute Level
Attribute 2	Attribute Level	Attribute Level
Attribute 3	Attribute Level	Attribute Level
Attribute 4	Attribute Level	Attribute Level
Attribute 5	Attribute Level	Attribute Level
Attribute 6	Attribute Level	Attribute Level
Attribute 7	Attribute Level	Attribute Level
Attribute 8	Attribute Level	Attribute Level

☐ Person A ☐ Person B

Introduction to conjoint experiment with random-chance option:

You already made 5 choices. In the following you will be presented with 5 more situations. *However, now you have the additional option to leave the selection to random chance.*

If you leave your decision to random chance, then, as in a coin toss, each of the two patients has an equal chance of being selected for ventilation or continued ventilation.

Conjoint experiment with random-chance option; every participant was given 5 of these pairs of profiles. Since the pairs were fully randomized, a generalized version is presented here (for a specific example see also fig. S2):

Please choose which person should be ventilated.
Please review the information on both patients thoroughly and make a recommendation.

	Person A	Person B
Attribute 1	Attribute Level	Attribute Level
Attribute 2	Attribute Level	Attribute Level
Attribute 3	Attribute Level	Attribute Level
Attribute 4	Attribute Level	Attribute Level
Attribute 5	Attribute Level	Attribute Level
Attribute 6	Attribute Level	Attribute Level
Attribute 7	Attribute Level	Attribute Level
Attribute 8	Attribute Level	Attribute Level

☐ Person A ☐ Person B ☐ Random Chance

Triage procedure ratings:

In a pandemic, such as COVID-19, many hospitals face situations where the demand for ventilators among patients in need will exceed the number of ventilators that are available. In that case, difficult decisions, just like the ones you just made, will need to be made about who should receive one of the scarce ventilators available. In the following, you see different procedures on how to determine the priority that patients have for being allocated a ventilator.

Please indicate how *morally fair* you find the following procedures.

	Not fair at all					Very fair
Maximize Prognosis:<br>Prioritize patients with the highest probability of survival after the treatment; i.e., treat those with the highest chance of recovery.	☐	☐	☐	☐	☐	☐
Youngest First:<br>Prioritize younger patients; i.e., treat those who have the most years of life left after overcoming the disease.	☐	☐	☐	☐	☐	☐
Life Quality after recovery:<br>Prioritize patients without any medical preconditions that would reduce quality of life after overcoming the disease; i.e., treat those with the highest quality of life they're likely to have after the treatment.	☐	☐	☐	☐	☐	☐
Random selection:<br>Ventilators should be allocated by random lottery; i.e., individual characteristics should not be considered.	☐	☐	☐	☐	☐	☐
First-come, first-served:<br>Prioritize patients who were first in line; i.e., treat those who arrived first at the hospital.	☐	☐	☐	☐	☐	☐
Sickest First:<br>Prioritize patients who suffer the most; i.e., treat those who are the worst off.	☐	☐	☐	☐	☐	☐
Benefit to others in the past:<br>Prioritize patients who have made relevant contributions to the benefit of others; i.e., treat those who have made sacrifices helping with the virus by having themselves vaccinated.	☐	☐	☐	☐	☐	☐
Benefit to others in the future:<br>Prioritize patients who are likely to make relevant contributions to the benefit of others; i.e., treat those who raise children.	☐	☐	☐	☐	☐	☐

Attention check question 2:

Please select "I fully agree” to indicate that you are paying attention to the questions.

☐	☐	☐	☐	☐	☐
I do not agree at all	I do not agree	I rather do not agree	I rather agree	I agree	I fully agree

Country of residence:

Which country you’re currently living in?
After you start typing recommendations will be shown. Please choose your country from the shown list.

Country: [ ]

Gender:

What describes you best?

☐ female
☐ male
☐ diverse

Year of birth:

What is your year of birth?

Year of birth: [ ]

Month of birth; drop-down list was shown:

Which is the month you were born in?

[⇕]

Education:

About how many years of education have you completed, whether full-time or part-time?

Please take into account all full- and part-time education (e.g., elementary, middle and high school) and higher education (e.g., college, apprenticeship), and convert the total duration of your school or training years into whole years.

[ ] years

Regression table

Table S3. Regression table for Fig. 1a) and Fig. 1c) in the main article, and Fig. S3 and Fig. S4 in this Appendix.

	Dependent variable:			
	Choice of a profile (dummy)			
	a)	c)	S3	S4
**Chance of survival (Ref.: 20% chance of survival)**				
50% chance of survival	0.186***	0.083***	0.152***	−0.124***
	(0.008)	(0.018)	(0.012)	(0.045)
80% chance of survival	0.299***	0.101***	0.254***	−0.281***
	(0.008)	(0.017)	(0.012)	(0.044)
**Age (Ref.: 25 years old)**				
50 years old	−0.115***	−0.070***	−0.094***	0.065**
	(0.008)	(0.012)	(0.009)	(0.028)
75 years old	−0.283***	−0.192***	−0.245***	0.113***
	(0.008)	(0.012)	(0.009)	(0.029)
**Disability (Ref.: Has no disability)**				
Has a disability	−0.012*	−0.005	−0.005	0.023
	(0.007)	(0.008)	(0.008)	(0.019)
**Weight (Ref.: Is not overweight)**				
Overweight	−0.030***	−0.025***	−0.026***	−0.004
	(0.007)	(0.008)	(0.008)	(0.019)
**Ventilation status (Ref.: Not ventilated yet)**				
Is already ventilated	0.001	−0.009	−0.021**	−0.050***
	(0.007)	(0.007)	(0.009)	(0.016)
**Vaccination status (Ref.: Is not vaccinated)**				
Is vaccinated	0.077***	0.055***	0.054***	0.008
	(0.007)	(0.008)	(0.009)	(0.016)
**Volunteer work (Ref.: Does not volunteer work)**				
Does volunteer work	0.038***	0.037***	0.031***	0.015
	(0.007)	(0.008)	(0.009)	(0.016)
**Children (Ref.: Has no children)**				
Has children	0.094***	0.071***	0.071***	0.007
	(0.007)	(0.008)	(0.008)	(0.017)
**Chance of survival x Maximize prognosis**				
50% chance of survival x Fair		0.154***	0.126***	0.070***
		(0.018)	(0.017)	(0.007)
80% chance of survival x Fair		0.316***	0.228***	0.138***
		(0.018)	(0.017)	(0.007)
**Age x Youngest first**				
50 years old x Fair		−0.087***	−0.115***	−0.042***
		(0.016)	(0.021)	(0.006)
75 years old x Fair		−0.175***	−0.208***	−0.091***
		(0.016)	(0.021)	(0.006)
**Disability x Life quality after recovery**				
Has a disability x Fair		−0.022	−0.020	−0.009**
		(0.014)	(0.014)	(0.005)
**Overweight x Life quality after recovery**				
Overweight x Fair		−0.014	−0.013	−0.007
		(0.014)	(0.014)	(0.005)
**Ventilation status x First come, first served**				
Is already ventilated x Fair		0.037**	0.045***	0.015***
		(0.015)	(0.013)	(0.004)
**Chance of survival x Sickest first**				
50% chance of survival x Fair		−0.029*	−0.032**	−0.009
		(0.016)	(0.016)	(0.006)
80% chance of survival x Fair		−0.079***	−0.091***	−0.025***
		(0.016)	(0.016)	(0.006)
**Vaccination status x Benefit in the past**				
Is vaccinated x Fair		0.090***	0.046***	0.021***
		(0.015)	(0.013)	(0.004)
**Volunteer work x Benefit in the past**				
Does volunteer work x Fair		0.004	0.015	0.007
		(0.015)	(0.013)	(0.004)
**Children x Benefit in the future**				
Has children x Fair		0.067***	0.066***	0.023***
		(0.014)	(0.014)	(0.004)
**Constant**	0.388***	0.460***	0.420***	0.561***
	(0.011)	(0.017)	(0.015)	(0.043)
Observations	19980	19980	19980	19980
R^2	0.132	0.155	0.148	0.162
Note:	*p<0.1; **p<0.05; ***p<0.01; Estimates of AMCEs on the probability of getting ventilation and interaction effects between attributes and procedure ratings. For readability, the main effects of procedure ratings are not displayed in the regression table. Clustered standard errors in parentheses.			

References

1. Storozuk A, Ashley M, Delage V, Maloney EA. Got bots? Practical recommendations to protect online survey data from bot attacks. The Quantitative Methods for Psychology. 2020;16(5):472–481. doi:10.20982/tqmp.16.5.p472.
2. Dunn AM, Heggestad ED, Shanock LR, Theilgard N. Intra-individual response variability as an indicator of insufficient effort responding: Comparison to other indicators and relationships with individual differences. Journal of Business and Psychology. 2018;33(1):105–121. doi:10.1007/s10869-016-9479-0.
3. Oppenheimer DM, Meyvis T, Davidenko N. Instructional manipulation checks: Detecting satisficing to increase statistical power. Journal of Experimental Social Psychology. 2009;45(4):867–872. doi:10.1016/j.jesp.2009.03.009.
4. Berinsky AJ, Margolis MF, Sances MW. Separating the shirkers from the workers? Making sure respondents pay attention on self-administered surveys. American Journal of Political Science. 2014;58(3):739–753. doi:10.1111/ajps.12081.
5. Hainmueller J, Hopkins DJ, Yamamoto T. Causal inference in conjoint analysis: Understanding multidimensional choices via stated preference experiments. Political Analysis. 2014;22(1):1–30. doi:10.1093/pan/mpt024.
